# Automated age estimation of young individuals based on 3D knee MRI using deep learning

**DOI:** 10.1007/s00414-020-02465-z

**Published:** 2020-12-17

**Authors:** Markus Auf der Mauer, Eilin Jopp-van Well, Jochen Herrmann, Michael Groth, Michael M. Morlock, Rainer Maas, Dennis Säring

**Affiliations:** 1 Medical and Industrial Image Processing, University of Applied Sciences of Wedel, Feldstraße 143, 22880 Wedel, Germany; 2 Department of Legal Medicine, University Medical Center Hamburg-Eppendorf (UKE), Butenfeld 34, 22529 Hamburg, Germany; 3 Section of Pediatric Radiology, Department of Diagnostic and Interventional Radiology and Nuclear Medicine, University Medical Center Hamburg-Eppendorf (UKE), Martinistr. 52, 20246 Hamburg, Germany; 4 Institute of Biomechanics M3, Hamburg University of Technology (TUHH), Denickestraße 15, 21073 Hamburg, Germany; 5 Radiologie Raboisen 38, Raboisen 38, 20095 Hamburg, Germany

**Keywords:** Age estimation, Magnetic resonance imaging, Knee, Machine learning, Convolutional neural networks

## Abstract

Age estimation is a crucial element of forensic medicine to assess the chronological age of living individuals without or lacking valid legal documentation. Methods used in practice are labor-intensive, subjective, and frequently comprise radiation exposure. Recently, also non-invasive methods using magnetic resonance imaging (MRI) have evaluated and confirmed a correlation between growth plate ossification in long bones and the chronological age of young subjects. However, automated and user-independent approaches are required to perform reliable assessments on large datasets. The aim of this study was to develop a fully automated and computer-based method for age estimation based on 3D knee MRIs using machine learning. The proposed solution is based on three parts: image-preprocessing, bone segmentation, and age estimation. A total of 185 coronal and 404 sagittal MR volumes from Caucasian male subjects in the age range of 13 and 21 years were available. The best result of the fivefold cross-validation was a mean absolute error of 0.67 ± 0.49 years in age regression and an accuracy of 90.9%, a sensitivity of 88.6%, and a specificity of 94.2% in classification (18-year age limit) using a combination of convolutional neural networks and tree-based machine learning algorithms. The potential of deep learning for age estimation is reflected in the results and can be further improved if it is trained on even larger and more diverse datasets.

## Introduction

The determination of certain age limits plays a crucial role in asylum applications, criminal proceedings, and professional youth sport. It can have important consequences for the persons in question. For example, special benefits apply for underage refugees [1, 2], specific laws are enforced to accused subjects [3], or exclusion from tournaments can occur for young athletes [4–6]. The retrieval of the chronological age is required whenever there is a lack of documentation or doubt about the alleged age [7–9].

The European Asylum Support Office (EASO) has presented guidelines on age estimation methods [10]. The recommendation is to perform the assessment of the chronological age using first non-medical and then medical methods. However, the results from non-medical methods, such as personal interviews or psychological assessments, are often inconclusive [11]. Hence, medical methods are necessary. These are based on the visual inspection of growth plate ossification of multiple long bones in medical images. The results from the inspection are then compared with reference data, e.g., Greulich and Pyle [12] or Tanner-Whitehouse 2 [13] hand/wrist atlases, to estimate the age of the subject. Other anatomical sites used for age estimation are the teeth [14], the clavicle [15], and the knee [16]. X-rays are frequently used for image acquisition as they offer a fast and inexpensive examination. But radiation exposure is considered “harm to the body” and should be used as a last option [10]. Moreover, the visual inspection is labor-intensive and subjective to the radiologist or expert conducting the analysis.

To overcome the disadvantages of X-ray-based age estimation, non-invasive, automated, and unbiased methods are required. In recent years, artificial intelligence (AI) has developed rapidly and gained popularity by enabling automated workflows and setting state of the art results in many medical applications [17, 18]. In age estimation, artificial neural networks (ANNs) seem to be particularly useful because they have the ability to independently extract and learn relevant information from data for a specific task [19]. Recent studies have delivered encouraging outcomes [20–30].

### Goal of this work

The purpose of the current work was to develop a method based on machine learning (ML) for the automated, computer-based, and non-invasive age estimation of male adolescents and young adults using three-dimensional (3D) magnetic resonance images (MRIs) of the knee.

In addition, the current work is dedicated to validating the results of a previous work [26] from 2018, but on a larger database, and then to developing a *new* robust and automated framework for age assessment based on knee MRIs. Furthermore, it can serve as a good comparison to a similar study by Dallora et al. [30] published in 2019, which also used deep learning and knee MRIs. Finally, the motivation to show that the promising results of Stern et al. [27]—who used deep learning for age estimation on the hand, collarbone, and teeth—are also suitable for another anatomical site, namely the knee.

## Materials

**Fig. 1:**
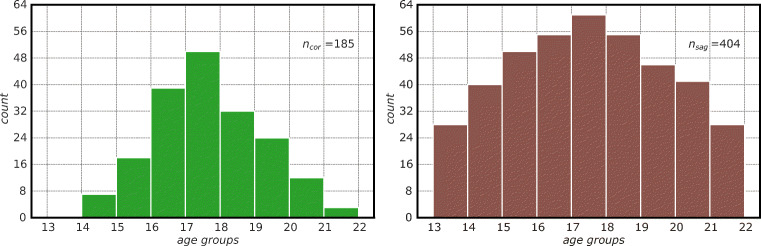
Age distribution for datasets DS_cor_ (left) and DS_sag_ (right). The imbalance in number of individuals per age group was mitigated by data augmentation (Section “Age estimation”)

The proposed automated and computer-based method for age estimation was applied and evaluated on two datasets: *n*_cor_ = 185 coronal and *n*_sag_ = 404 sagittal 3D MRIs of the knee. The coronal dataset DS_cor_ was acquired from two prospective studies [16] and [31] and included MRIs of 79 male Caucasian subjects in the age range of 14.41 and 21.66 years (mean ± sd: 17.60 ± 1.53 years; minors: 61.62%). The sagittal dataset DS_sag_ was acquired from the above-mentioned prospective studies and expanded with retrospective data. It included MRIs of 297 male Caucasian subjects in the age range of 13.00 and 21.83 years (mean ± sd: 17.42 ± 2.28 years; minors: 57.92%). The chronological age of the subjects was calculated as the difference between the birth and MRI acquisition dates. The age distributions for both datasets are shown in Fig. 1.

To attain a homogeneous study population, the following criteria were established for the subject recruitment: Caucasian, male, middle to high socio-economic status, raised in Hamburg (Germany) or surroundings, between 13 and 21 years of age, and no chronic diseases or severe bone injuries.

### MRI protocol

MR images were retrieved with six different MRI scanners, four with 3.0T and two with 1.5T field strength, and included sequences in both coronal and sagittal orientation. The protocols used were T1-weighted TSE (Turbo Spin Echo) and T1-weighted SENSE (SENSitivity Encoding). The coronal sequences had the following specifications: TR 600–850 ms, TE 10–20 ms, flip angle 90^∘^. The sagittal sequences were executed with the parameters: TR 590–5000 ms, TE 10–70 ms, flip angle 90–150^∘^.

The acquired coronal MRIs had a resolution in voxels which ranged between 512 × 512 × 24 and 800 × 800 × 41 and a voxel size in mm^3^ extending from 0.19 × 0.19 × 2.2 until 0.49 × 0.49 × 4.9. The sagittal images had resolutions of 512 × 512 × 24 voxels–1050 × 1050 × 50 voxels and the voxel sizes were in the range of 0.17 × 0.17 × 2.2 mm^3^ to 0.49 × 0.49 × 5.2 mm^3^.

### Additional subject-related data

In addition to the MRI data, additional *subject-related data* were collected for subjects in DS_cor_. This data was composed of anthropometric measurements (AM) and the ossification stages (OS) of the growths plates of the knee. AM included the weight, standing and sitting height, and lower leg length (LLL). The weight was measured with a standard body scale device, the standing and sitting height were acquired with an anthropometer corresponding to the standardized measuring length [32, 33], and the LLL was measured using an anthropometric device [34, 35].

For the OS, the three-stage system by Jopp et al. [16] was selected and applied to the epiphyses of the distal femur (DF), proximal tibia (PT), and proximal fibula (PF). The stages of the individual bones were also summed to generate the *score of the knee joint* (SKJ), similar to Galić et al. [36]. A statistical analysis of the classification system on a sub-sample of DS_cor_ can be found in a previous study [31].

## Methods

**Fig. 2:**
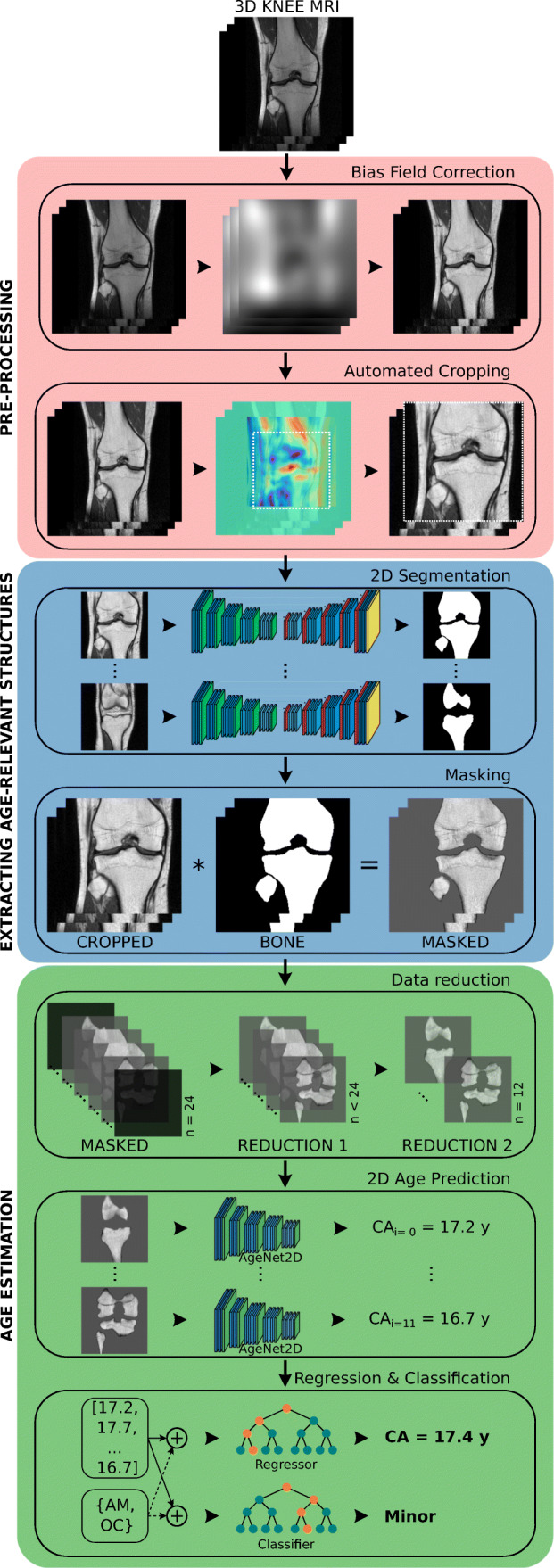
Proposed solution for automated age estimation

The proposed approach of the current work for automated age estimation is presented in Fig. 2. The workflow is composed of three major parts: *Pre-processing* (Section “Pre-processing”) of the 3D knee MRIs, *Extracting age-relevant structures* (Section “Extracting age-relevant structures”) in the images, and finally, *Age estimation* (Section “Age estimation”) based on the pre-processed images and subject-related data acquired from the study participants.

The approach resembles the method presented in a previous work [26] but introduces the following important novelties regarding data, pre-processing, and age estimation:

- Larger DS_cor_ and a newly acquired DS_sag_
- A more robust automated cropping approach as part of the pre-processing, capable of cropping both coronal and sagittal knee MRIs
- Improved selection of the most informative images from the MRI volumes
- Age-stratified image augmentation to account for imbalance in the age distribution
- New and robust deep learning model architecture for age estimation on knee MRI
- Classification of minors and adults
- A *repeated* stratified fivefold cross-validation to account for the stochastic nature of deep learning

More details can be found in the following sub-sections of this manuscript. For a complete and thorough presentation of all developed and tested methods, especially the design and testing of various machine learning algorithms and deep learning network architectures for age estimation, see [37].

### Pre-processing

*Bias field correction* (BFC) was a necessary step to correct low-frequency intensity non-uniformities in the MRIs caused by inhomogeneities in the magnetic field (Fig. 2—salmon-colored box). N4ITK [38], the de facto standard in the medical image processing field, was used for this purpose. Before the application of N4ITK, all images were downsampled to a common in-plane resolution of 448 × 448 pixels. This step was essential to address the issue of different image resolutions—caused by the use of different MRI scanners and sequences—and to accelerate the processing time of the N4ITK algorithm.

**Fig. 3:**
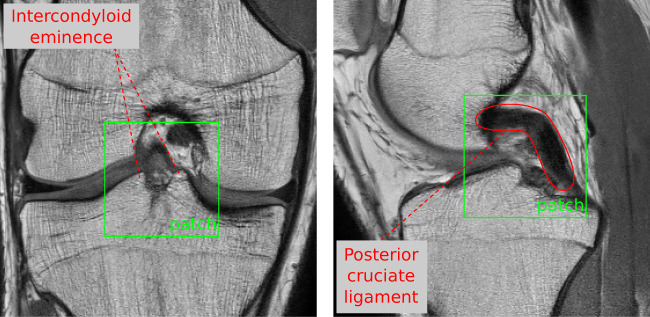
Characteristic regions defined in coronal (left) and sagittal MRIs (right) for the template matching algorithm of the automated cropping

*Automated cropping* was used to generate standardized volume of interests (VOI) from the bias-field-corrected images due to differences in leg position and selection of the field of view (FOV) during MR examination (Fig. 2—salmon-colored box). For this purpose, an approach based on *template matching* (e.g., [39, 40]) was developed. It computed the best correlation between a predefined characteristic region and equally sized regions in the image in a sliding-window fashion. The characteristic region defined for coronal MRIs was a small *patch* showing the tubercles of intercondylar eminence and for sagittal MRIs, a region around the posterior cruciate ligament (PCL) (Fig. 3). Differences in in-plane resolution, resulting from data acquisition with different MRI scanners, were taken into account by resampling the pre-defined patches accordingly. Finally, the standardized VOI was extracted using the point of the highest correlation as the center and then extending 130 mm in *x* and *y* directions and along the entire depth of the MRI volume.

### Extracting age-relevant structures

After pre-processing the acquired images, there were two options to train a deep learning network for age estimation: (a) to feed the pre-processed and almost “raw” MRIs or (b) to feed only age-relevant structures such as bones and growth plates to simplify the age estimation problem from appearance information. Both options were tested using a convolutional neural network (CNN) described in Section “Age estimation.” In option (a), the training for age estimation led to an underperforming learning progression (Fig. 5). The error of the network for unknown data (orange line) did not improve significantly after hundreds of training cycles, while the error on the data on which the network learned (blue line), i.e., the training data, decreased steadily. The differences between the error curves is known as a *generalization gap* and indicates that the training data are not representative and therefore the model cannot generalize to new data.

To be able to test option (b), the bones and growth plates had first to be extracted from the images. As in the previous work [26], training a CNN to detect these age-relevant structures proved to be an excellent choice (Fig. 2—blue box).

For this purpose, ground truth structures had to be labelled in the original MRIs in a first step. A sub-set of 100 coronal and 25 sagittal ground truth segmentation maps of the bones were generated using semi-automatic region growing and manual correction by a researcher (MAdM) well experienced in the analysis of 3D knee MRIs and subsequently approved by a radiologist (JH).

To solve the bone segmentation task, the standardized VOIs from the pre-processing had to be further processed to be suitable for the CNN. Each slice was first *downsampled* to a size of 224 × 224 pixels and then *normalized* to transform all pixel intensities to a similar range of values. The *standard score* was used to normalize the input to zero mean and a standard deviation of one.

The final input to the CNN was standardized, downsampled, and normalized two-dimensional image slices of an MRI volume. The architecture of the CNN resembled U-Net [41] and was adapted for the bone segmentation in knee MRIs. This network type is known as *autoencoder*. It first compresses the input to learn features at different scales and then restores the input to predict a segmentation map in the original size. The final architecture for the segmentation task was identical to a previous work [26] (please refer to the paper for further details).

For the CNN-based segmentation a total of 100 three-dimensional coronal MRIs and their corresponding ground truths were available. The data was randomly split into the training set (70%), the validation set (15%), and the test set (15%). To increase the robustness of the network, the size of the training set was virtually increased via *augmentation*. In contrast to the previous work [26], training images were not only translated but additionally rotated, flipped, and cropped using three different VOI sizes.

The training setup, i.e., optimizer, learning rate, loss function, metrics, batch size, epochs, and early stopping, matched the one in [26]. Differently, CNN-based segmentation was not only applied to coronal but to sagittal MRIs as well. To reduce the number of ground truths for training, the CNN for sagittal data was initialized with the weights learned from the coronal model and then retrained for 5 epochs.

Finally, the segmentation maps predicted by the CNNs were used to extract age-relevant structures from the standardized VOIs, i.e., bones and growth joints, by *masking* (Fig. 2—blue box). This step generated the desired input for the age estimation part described next.

The extraction of age-relevant structures as in option (b) led to better and more stable learning curves (Fig. 5) than option (a), since the learned knowledge about bone segmentation could be incorporated into the age estimation network using *transfer learning*.

### Age estimation

The proposed age estimation part of the current work was composed of three parts (Fig. 2—green box): part one was the *data reduction* of the masked MRIs, part two was the *2D age prediction* based on the reduced images using a CNN, and part three was the ultimate *age regression and majority classification* based on the CNN age predictions and the subject-related data using machine learning algorithms. For simplicity, the pipeline is explained for coronal images in this sub-section but was performed for sagittal MRIs as well.

#### Data reduction

Due to empty image slices in the masked MRIs and to further simplify the age estimation problem, the image volumes per subject were reduced in two phases. *Reduction 1* removed image slices with no or sparse bone content, only keeping slices with more than 2% bone information related to the total size of the volume. In *Reduction 2*, all MRIs were further reduced to a predefined minimum of 12 slices. This final reduction ensured a balance of data per subject, similar bone information per slice, and the possibility to train a neural network based on the 3D context. Reduction 2 was implemented by first computing a bone-amount-per-slice distribution and then evenly selecting image slices along the volume with a reference at the center of gravity (CoG) of the distribution.

#### 2D age prediction (AgeNet2D)

Prior to designing and training a neural network for age estimation, the 185 coronal, masked, and reduced MRI volumes (*n*_2*D*_ = 185 × 12 = 2220) were split into three new sub-sets: training (≈ 63%), validation (≈ 18%), and test (≈ 19%) sets. *This age-stratified random split* ensured that all sets had a similar age distribution. Moreover, it was assured that the training set included the entire age range (14–21 years) of the coronal data, such that the model could effectively predict any age in that range.

Due to the imbalance of the age groups in DS_cor_, 2D image augmentation was applied only to the images of the less represented age groups of the training set. The types of augmentation included the generation of two additional FOVs per image, horizontal translation, and rotation. This increased the number of training samples from 1392 to 2412 two-dimensional images.

*AgeNet2D* is the CNN that was developed as part of the age estimation task in the current work. It was created as a variant of the contracting path, i.e., the encoder, of the segmentation network designed in the previous work [26]. The encoder is the part of a CNN that compresses the input images.

AgeNet2D included several different types of layers such as Convolution (*conv*), Exponential Linear Unit (*elu*), Batch-Normalization (*bn*), Dropout (*do*), Max-Pooling (*mp*), Global Average Pooling (*gap*), Fully Connected (*fc*), and Linear Activation (*lin*).

Convolution is the primary operation in CNNs, which convolves a set of small learnable filters, i.e., kernels, across the entire width and height of the image. The outputs of a convolutional layer are known as *feature maps*. ELU [42] is an activation function appended to a convolution operation to introduce a non-linearity into the network. Batch Normalization [43] is a popular technique that standardizes the inputs of a layer which results in more stable and faster training and reduced generalization error due to its regularization effect [44]. Dropout [45] randomly drops convolutional kernels and their connection to other layers during training—controlled by the dropout rate *p*—and enables the network to learn independent features [45, 46]. Thus, Dropout acts as an effective regularization measure against overfitting. Max-Pooling reduces the size of features maps, allowing the network to effectively learn features at different scales. Furthermore, it has the benefit of decreasing the amount of calculations and lowering the risk of overfitting [47]. GAP [48, 49] and FC layers are often used at the end of CNNs to reduce the dimensionality of the feature maps before the output of the network is generated via a final activation function, such as a Linear Activation.

**Fig. 4:**
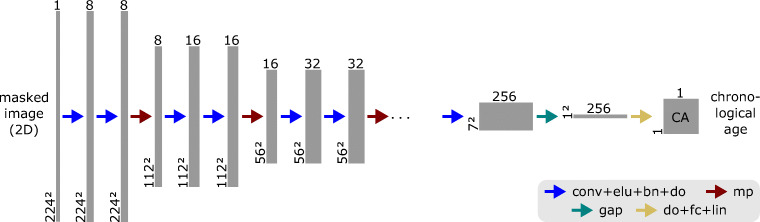
*AgeNet2D*—the CNN developed for age estimation—uses 2D masked knee MR slices (only bone and growth plates) and predicts the chronological age of a subject. The network compresses the input from 224^2^ to 7^2^ pixels using Convolution (*conv*) and Max-Pooling (*mp*) operations to compute features at different scales. Exponential Linear Unit (*elu*) is an activation function, Batch-Normalization (*bn*) standardizes layer inputs, Dropout (*do*) randomly drops convolutional kernels, Global Average Pooling (*gap*), and Fully Connected (*fc*) are further layers that condense the features and finally, the Linear activation (*lin*) layer regresses the chronological age (adapted from [37])

The final architecture of AgeNet2D (Fig. 4) resulted from a comprehensive *grid search*, which tested different number, order, and combinations of layers as well as different values of hyperparameters (e.g., dropout rate or kernels).

It was composed of five “Down Blocks” with the following sequence of layers: two sets of “3 × 3 conv → elu → bn → do (*p* = 0.1 × *d*)” followed by one “2 × 2 mp ”. The *mp*-layer halved the feature maps’ dimension at each network depth *d*, thus compressing the input image from 224^2^ to 7^2^ pixels. The last part of the network contained the layers sequence: “gap → do (*p* = 0.5) → fc → lin.” The *gap*-layer was used to reduce the dimensionality of the features maps from 7 × 7 × 256 to 1 × 1 × 256 by averaging each feature map. Finally, a sequence of *do* (*p* = 0.5), *fc*, and *lin* layers was used to regress the chronological age from the last 256 features. The entire network contained a total of 1.18 million trainable parameters.

Due to the complexity of the age regression problem, the number of kernels per convolutional layer had to be doubled with network depth, starting from 8 up to 256 kernels, in order to find correlating features in the images. In addition, the initial dropout rate of *p* = 0.1 in the Down Blocks was multiplied with the network depth *d* to account for the increasing number of parameters.

After defining the final architecture, the weights and biases of the network layers, i.e., the trainable parameters, had to be initialized. The options were either to assign random values (the standard approach) or to initialize the parameters with “pre-trained values” learned from a different but similar problem. The latter was possible because a neural network had already been trained on knee MRIs, but for the segmentation problem. Both cases were tested, but the use of transfer learning led to more stable training processes and better results.

To enable this transfer of knowledge, the segmentation network of the previous work [26] had to be slightly modified to correspond to the layer sequences as shown in Fig. 4, and retrained. Then the learned parameters from the encoder were used to initialize the weights and biases of AgeNet2D.

After initializing the network, the training process was started to learn the best possible 1.18 million parameters for age estimation from knee MRIs. To achieve this, an optimization algorithm must iteratively change parameter values to minimize the error or loss of the task. Using the Adam optimizer [50] and the loss function of the mean squared error (MSE) proved to be a good choice for solving the given problem. MSE is defined as follows:

1 $$ \text{MSE} = \frac{1}{n} \sum\limits_{i=1}^{n} (y_{i} - \hat{y}_{i})^{2}, $$

where *n* are the number of samples, *y*_*i*_ the true age of the *i* th subject, and $\hat {y}_{i}$ the predicted age by the network. To control how much the optimizer modifies the parameters depending on the loss after a certain number of training samples, i.e., batch, the *learning rate* had to be set. The default value of Adam is 0.001, but due to initialization with pre-trained parameters, the learning rate was reduced to 0.0001 to ”fine tune” the CNN for the age estimation task. The optimal *batch size*, after which a weight update of the network had to be performed, was 16, and resulted from several test runs. The last hyperparameter that was set was the number of training cycles or *epochs*. Due to the complexity of the problem and the low learning rate, AgeNet2D was trained over 1000 epochs.

Finally, after CNN training, all pre-processed, masked, and reduced 2D MRI could be fed into the network to predict chronological age.

#### Regession and classification

To regress the final age of a subject based on knee MRIs, the 12 age predictions of AgeNet2D had to be combined. Instead of taking the average or median or even using the minimum-age concept [51], machine learning algorithms (MLAs) were trained on the 12 age predictions of AgeNet2D to finalize the age estimate per subject. This approach not only led to more accurate results but also enabled the integration of the subject-related data in the age estimation process of DS_cor_ (Fig. 2—last part of the green box). For DS_sag_, this additional data was not available.

Classification between minors and adults is an essential task in forensic age estimation. Consequently, this problem was also investigated. The straightforward approach would be to use the regression estimate for classification. However, classification based on initial tests showed fewer errors when an MLA based on 12-age predictions (+ subject-related data) was trained to separate minors from adults. It was particularly useful for more accurate classification of subjects near the 18-year age limit.

The MLAs trained for this work included Support-Vector Machines (SVM), Linear Regression (LR), K-Nearest Neighbors Classifier (KNC), and tree-based algorithms such as Random Forests (RF) and Gradient Boosting (GB).

### Model evaluation

The evaluation of the trained models was performed on the *test set*, i.e., on the fraction of the data from which the model did not learn. To obtain a robust and unbiased estimate of model performance, a repeated stratified *k*-fold cross-validation (CV) with *k* = 5 folds was performed.

During the generation of the fivefold, it was ensured that each test set was unique. The stratification also guaranteed that the folds and sets had a similar age distribution. Finally, due to the stochastic nature of depth learning and most machine learning algorithms, the evaluation was repeated ten times for each fold. This provided a more reliable assessment of the performance of each model.

The *mean absolute error* (MAE) was the principal metric used to evaluate the regression models:

2 $$ \text{MAE} = \frac{1}{n} \sum\limits_{i=1}^{n} \lvert(y_{i} - \hat{y}_{i})\rvert, $$

where *y*_*i*_ is the true chronological age of the *i* th subject, $\hat {y}_{i}$ the prediction by the model, and *n* the total number of subjects. Further metrics tracked during the evaluation were the standard deviation of the absolute error, and the 95% confidence interval of the mean (95% CI).

For the classification of the 18-year age limit, the accuracy, the sensitivity, and the specificity were the main evaluation metrics:

3 $$ \begin{array}{@{}rcl@{}} \text{Accuracy} &=& \frac{\text{TP} + \text{TN}}{\text{TP} + \text{TN} + \text{FP} + \text{FN}}, \end{array} $$

4 $$ \begin{array}{@{}rcl@{}} \text{Sensitivity} &=& \frac{\text{TP}}{\text{TP} + \text{FN}}, \end{array} $$

5 $$ \begin{array}{@{}rcl@{}} \text{Specificity} &=& \frac{\text{TN}}{\text{TN} + \text{FP}} \end{array} $$

with TP as true positives, TN true negatives, FP false positives, and FN false negatives. All minors, i.e., below 18 years of age, were defined as TPs. Thus, the accuracy represents the number of correct predictions (whether minor or adult) over the total samples of the test set. The sensitivity corresponds to the number of minors that were correctly classified as such, while specificity is the number of adults that were correctly classified. The metrics range between 0 and 1, where values closer to 1 represent better performance.

Another metric used to compare the performance of different models was the *Area Under the Curve* (AUC) score. It represents the area under the *Receiver Operating Characteristic* (ROC) curve and indicates the capacity of the model to discriminate between minors and adults. The score can take any value between 0 and 1. A perfect classifier has a score of 1 while a “naive” classifier has 0.5. The latter one would simply classify by chance.

#### Statistical reference

To confirm the added value of the rather complex age estimation framework presented in the current work, the trained models were compared with the *best available guess* [52].

For regression, the best available guess was defined as the mean age of the training set distribution.

In contrast, the best available guess for classification was to predict all subjects as minors, i.e., a “naive” classifier. This resulted in a sensitivity of 1.0, a specificity of 0.0, and an AUC score of 0.5.

## Results

**Fig. 5:**
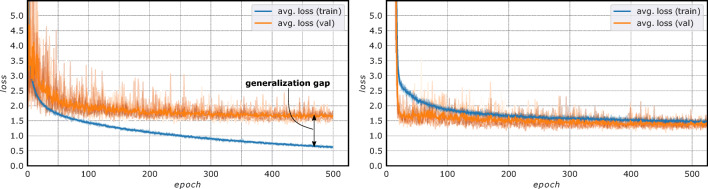
Training progression of AgeNet2D using “raw” or “unsegmented” knee MRIs (left) vs. using *masked* images (right). The mean squared error *loss* is plotted for the known data (blue), i.e., the training set, and the unknown data (orange), i.e., the validation set, over multiple epochs. Using masked knee MRIs improved training progression and avoided the *generalization gap* created by CNN training on “raw” images. This supports the assumption that the age estimation problem could be simplified by extracting age-relevant structures from knee MRIs (adapted from [37])

Simplifying the age estimation problem by extracting age-relevant structures in knee MRIs was successful (Fig. 5). The training progression improved and the model was able to generalize on unseen data using masked knee MRIs in comparison to using “raw” images. This was verified quantitatively by evaluating the two scenarios—“raw” or *un* segmented vs. *masked* MRIs—on a single fold of DS_cor_. Age predictions with a CNN improved markedly from an average MAE of 0.97 ± 0.84 years (“raw”) to 0.81 ± 0.65 years (masked) on coronal knee MRIs when trained several times on a single fold.

### Model performance on regression

The results for age regression based on AgeNet2D—with and without MLAs—using the underlying DS_cor_ and DS_sag_ datasets are enlisted in Table 1. The results for each dataset are divided into three scenarios: best available guess, repeated CV results (*rep*.), and the best CV results across all five folds (*best*).

**Table 1:** *Age regression* performance on the test sets in a (repeated) fivefold cross-validation (CV) using masked knee MRIs and subject-related data (srd). Metrics are presented in years and include the mean absolute error (MAE), the standard deviation (SD), and the 95% confidence interval of the mean (95% CI)

CV	Model	MAE ± SD	95% CI
DS_cor_			
-	Best available guess^a^	1.63 ± 0.99	[1.59; 1.67]
rep.^b^	AgeNet2D	0.81 ± 0.65	[0.80; 0.83]
	AgeNet2D+ MLA^c^	0.71 ± 0.55	[0.71; 0.72]
	AgeNet2D+ MLA_srd_	0.73 ± 0.55	[0.71; 0.74]
best^d^	AgeNet2D	0.79 ± 0.62	[0.74; 0.83]
	AgeNet2D+ MLA	0.67 ± 0.49	[0.62; 0.72]
	AgeNet2D+ MLA_srd_	0.69 ± 0.47	[0.63; 0.74]
DS_sag_			
-	Best available guess	1.93 ± 1.20	[1.90; 1.96]
rep.	AgeNet2D	0.92 ± 0.73	[0.91; 0.94]
	AgeNet2D+ MLA	0.81 ± 0.62	[0.79; 0.83]
best	AgeNet2D	0.89 ± 0.70	[0.82; 0.96]
	AgeNet2D+ MLA	0.79 ± 0.57	[0.67; 0.90]

^a^Mean age of the training set

^b^Repeated cross-validation for all five folds

^c^Machine learning algorithm

^d^Best cross-validation for all five folds

**Fig. 6:**
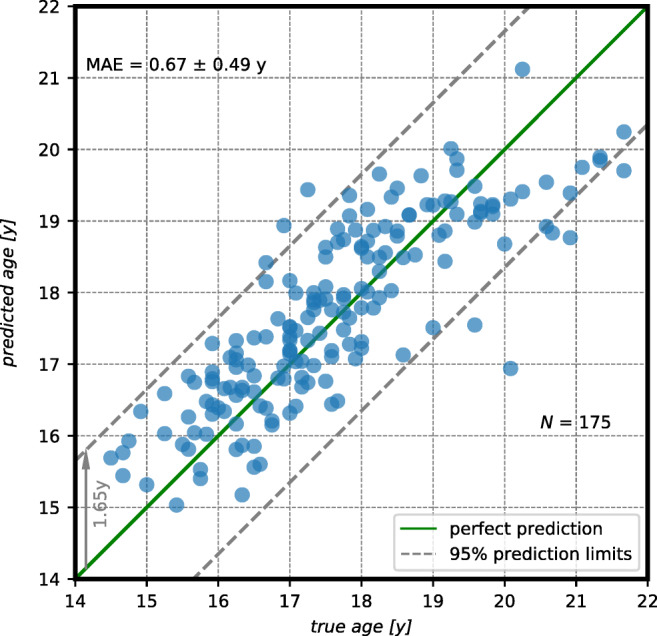
Predicted vs. true chronological age of test subjects from all five folds (*n* = 35 ∗ 5 = 175) using AgeNet2D followed by an Extremely Randomized Trees Regressor (ETR) trained on masked coronal knee MRIs. The green central line highlights a perfect prediction, while the two parallel grey lines encompass 95% of the model predictions

All regression models trained on DS_cor_ outperformed the best available guess (1.63 ± 0.99 years) of the training sets. In addition, the introduction of an MLA to finalize the age prediction improved all metrics by more than 10% compared to the use of AgeNet2D as the sole predictor. The inclusion of the subject-related data did not boost the performance of the models but rather hurt the MAE. Overall, the best-performing regression model across all folds for DS_cor_ combined AgeNet2D with an *Extremely Randomized Trees Regressor* (ETR) as MLA and neglected the subject-related data. It achieved a MAE of 0.67 ± 0.49 years and had a 95% CI of [0.62; 0.72]. The predicted ages by the model over all folds are plotted over the true chronological ages in Fig. 6.

The best available guess on DS_sag_ yielded inferior metrics compared to that on DS_cor_ due to a larger dataset, a wider age range, and a more uniformly distributed training set. Similar to the coronal results, the regressors on sagittal MRIs outperformed the results using the best available guess (1.93 ± 1.20 years) of the training set significantly (*p* < 0.001). The introduction of MLAs improved regression metrics by a large extent. The best-performing model across all folds was AgeNet2D followed by an ETR as MLA with a MAE of 0.79 ± 0.57 years and a 95% CI of [0.67; 0.90]. Although the metrics were inferior compared to the ones on DS_cor_, the sagittal models were based on a broader and larger population.

### Model performance on classification

All listed models achieved above 80% in accuracy, sensitivity, specificity, and AUC on majority classification (Table 2). The best-performing model on DS_cor_ surpassed 89% in accuracy and combined AgeNet2D and a *Random Forests Classifier* (RFC) as MLA. The model using MLA without subject-related data had a slightly higher average sensitivity and AUC in comparison to the MLA using it. This is the desired outcome to correctly classify more minors.

**Table 2:** Performance on *majority classification* (≥ 18 years) on the test sets in a (repeated) fivefold cross-validation (CV) using masked knee MRIs and subject-related data. Metrics are presented in percentage and include accuracy (Acc.), sensitivity (Sens.), specificity (Spec.), and area under the receiver operating characteristic curve (AUC)

CV	Model	Acc.	Sens.	Spec.	AUC
*DS*_cor_					
-	Best available guess^a^	49.3	100.0	0.0	50.0
rep.^b^	AgeNet2D+ MLA^c^	85.7	86.4	84.6	90.8
	AgeNet2D+ MLA_srd_	83.5	81.4	87.1	89.6
best^d^	AgeNet2D+ MLA	89.1	89.1	89.2	92.5
	AgeNet2D+ MLA_srd_	89.7	88.2	92.3	92.0
*DS*_sag_					
-	Best available guess	52.3	100.0	0.0	50.0
rep.	AgeNet2D+ MLA	87.5	88.4	86.1	94.3
best	AgeNet2D+ MLA	90.9	88.6	94.2	94.4

^a^Predict minors for the entire training set

^b^Repeated cross-validation for all five folds

^c^Machine learning algorithm

^d^Best cross-validation for all five folds

**Fig. 7:**
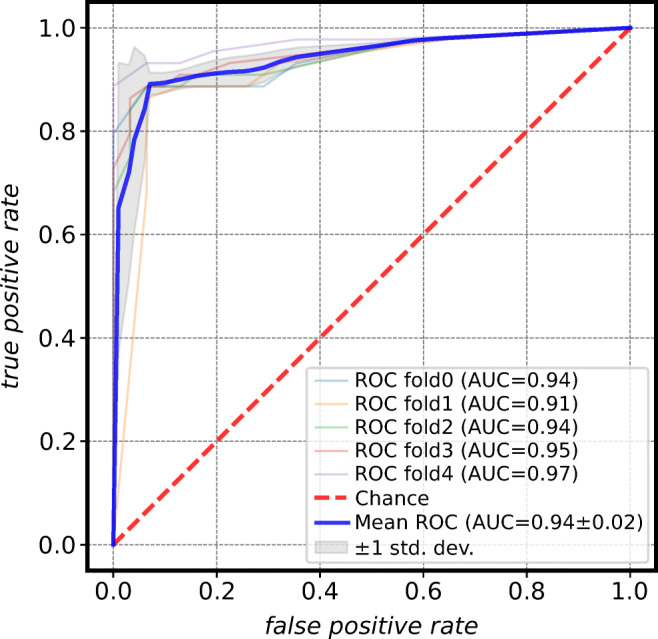
Receiver operating characteristic (ROC) curve for the best-performing model on majority classification (≥ 18 years). The Random Forests Classifier (RFC) attained an accuracy of 90.9%, a sensitivity of 88.6%, a specificity of 94.2%, and an area under the ROC curve (AUC) of 94.4% averaged over five folds. The high mean AUC indicates that a shift of the threshold can further improve the sensitivity at the expense of the specificity (adapted from [37])

Surprisingly, the models trained on DS_sag_ had better classification metrics although the regression results were inferior in comparison to DS_cor_. Once more, an RFC delivered the best average metrics when trained on AgeNet2D’s age predictions from sagittal MRIs. It attained an accuracy of 90.9%, a sensitivity of 88.6%, a specificity of 94.2%, and an AUC of 94.4% over all folds. The ROC curve of the model indicates that it has the potential to increase its sensitivity, at the cost of specificity, to increase the amount of correctly classified minors (Fig. 7).

## Discussion

The proposed method enabled a non-invasive, computer-based, and fully automated solution for age estimation based on an actual and homogeneous population. In contrast, current methods in practice rely on labor-intensive and subjective visual assessments of the growth plate ossification stages. This assumes that the stages provide sufficient criteria to distinguish minors from adults. Contradictory results can be observed in studies claiming that the staging system used allows for separation between minors and adults [53–55] while other studies claim the opposite [16, 31, 56, 57]. Further information and differences of studies using MRI for age estimation can be found in the systematic review by De Tobel et al. [58].

### Principal findings

The idea to use bone segmentation to simplify age estimation based on appearance information was successful. AgeNet2D was able to find correlating features with age in the images in a more robust and generalizable manner when using masked instead of “raw” knee MRIs. In [28], the authors had similar findings when cropping age-relevant structures from hand MRIs instead of using the whole image volume as input of a CNN. The benefit of executing bone segmentation *prior* to age estimation in the current work was the possibility to transfer knowledge from one problem to the next. Stern et al. [21] and Dallora et al. [30] were also successful in improving age prediction by using *transfer learning*.

Age predictions by AgeNet2D—based on 2D masked knee images—were effectively combined to a final age per subject using ML-based algorithms to enhance age regression further. An alternative to avoid this two-step age estimation would be to use 3D CNNs on the knee MRI volumes. First tests on both datasets DS_cor_ and DS_sag_ showed inferior results in comparison to the combination of CNNs and ML-based regressors. Nevertheless, a 3D-CNN has the capacity to collect more contextual information from the MRI volume and this potential should be evaluated in future work.

When comparing the age regression methods of the current work, the coronal MRI-based models were superior to the models on sagittal MRI. The difference could be related to a larger sagittal dataset with a broader age range and a more uniform age distribution of DS_sag_, which could have introduced a higher possibility of variance and outliers.

Regarding the classification task on the 18-year age limit, comparable results were attained using coronal or sagittal knee MRIs. The high AUC of 0.94 for the classifiers on sagittal data shows the capacity of this model to differentiate between adults and minors. A change of threshold can improve the amount of correctly classified minors which is a more favorable outcome in practice.

The inclusion of subject-related data, namely ossification stages and anthropometrics data, in the ML-based regressors and classifiers did not increase age estimation performance. In the classification task, it even slightly hurt the outcome. The limited contribution of anthropometric data when used in conjunction with MR images was also observed in [52]. Contrary, the inclusion of ossification stages positively contributed to the performance on age estimation in [21]. However, the data was not included in an MLA but used to pre-train a CNN, to achieve a better initialization of the network. Training a CNN on ossification stages rather than segmentation could be a viable alternative to the method developed in the current work.

### Comparison to similar studies

Similar studies in the field of automated age estimation are the works by Stern et al. [21, 27, 28, 59, 60] and Dallora et al. [30]. Both research groups have developed and analyzed methods for age estimation based on machine learning (incl. deep learning) as well. Further resemblance to the current work is the MRI modality, the number of samples, and the population, which included males in similar age ranges from 13 to 25 years (Table 3). While Dallora et al. also acquired MRIs of the *knee*, Stern et al. have focussed on different anatomical sites: *hand*, *clavicle*, and *wisdom teeth*.

**Table 3:** Comparison of age regression performance between the current work and other studies using automated and MRI-based methods. Techniques used include convolution neural networks (CNNs), machine learning algorithms (MLAs), gray-level variations (GLV), and principal component analysis (PCA). The metrics are presented in years and include the mean absolute error (MAE) and the standard deviation (SD)

Study	*N*	Gender	Age range (years)	Anatomical site	Technique	MAE ± SD
Pröve et al. [26]^a^	145	Male	14–21	Knee	CNN+ MLA	0.48 ± 0.32
Current work^b^	185	Male	14–21	Knee	CNN+ MLA	0.67 ± 0.49
Current work^c^	404	Male	13–21	Knee	CNN+ MLA	0.79 ± 0.57
Dallora et al. [30]	221 (181)	Male (female)	14–21	Knee	CNN	0.79^d^
Stern et al. [59]	56	Male	13–19	Hand	MLA	0.85 ± 0.58
Stern et al. [60]	132	Male	13–20	Hand	MLA	0.82 ± 0.56
Stern et al. [21]	103	Male	13–24	Hand, teeth, clavicles	CNN	1.14 ± 0.96
Stern et al. [27]	322	Male	13–25	Hand, teeth, clavicles	CNN	1.01 ± 0.74
Stern et al. [28]	328	Male	13–25	Hand	CNN	0.82 ± 0.65^e^
Saint-Martin et al. [61]	80 (80)	Male (female)	8–25	Ankle	GLV+PCA	-

^a^Previous work as a proof of concept *without* *k*-fold cross-validation

^b^Results of current work using *coronal* knee MRIs

^c^Results of current work using *sagittal* knee MRIs

^d^SD was not indicated in the paper, and the metric omits the MAE of 1.37 years for the 21-year-old age group

^e^Metric only holds for subjects ≤ 18 years

From Table 3, it is visible that the models from the current work outperform the ones of Stern et al. and Dallora et al. in age regression on the basis of MAE. However, the differences can be caused by several factors such as a different age distribution, anatomical sites, sample number, pre-processing, and CNN architecture. The even lower MAE from a previous work [26] is less comparable because it was a proof of concept and no cross-validation was performed. A common result of these studies is the improvement in age regression through CNN *pre-training*. In the current work, the weights were initialized using the knowledge from the segmentation task. Stern et al. [21] pre-trained their CNN using information about the maturation of the growth plates by radiological assessment. In contrast, the models of Dallora et al. [30] were pre-trained on ImageNet [62], a large database of roughly 3.2 million images of animals, vehicles, etc.

Besides age regression, the studies mentioned above delivered results on the classification task as well. The metrics are markedly similar to the ones of the current work (Table 4). Stern et al.’s higher specificities and lower sensitivities could be caused due to the larger amount of adults in their dataset. Nonetheless, the highlight is their excellent AUC of 0.98 in [27]. Dallora et al. has the highest sensitivity among all studies but does not provide values on accuracy and AUC. Another automated approach using MRI as modality which evaluated the classification task is the work by Saint-Martin et al. [61]. For their male dataset of 80 individuals, they obtained a low sensitivity of 69% but a very high specificity of 98%. In contrast to the current work, they investigated a different anatomical site (distal tibia) and had a wider age range (8–25 years).

**Table 4:** Comparison of majority classification (≥ 18 years) performance between the current work and other studies using automated and MRI-based methods. Besides accuracy, sensitivity, and specificity the area under the curve (AUC) represents the capacity of a model to discern between minors and adults. Metrics are presented in percentage. An overview of the population, datasets, and methods of the studies can be found in Table 3

Study	Minors	Accuracy	Sensitivity	Specificity	AUC
Current work^a^	49.3	89.7	88.2	92.3	92.0
Current work^b^	52.3	90.9	88.6	94.2	94.4
Dallora et al. [30]	47.1^c^	-	90.4^c^	95.6^c^	-
Stern et al. [21]	42.7	91.3	88.6	93.2	-
Stern et al. [27]	41.6	90.7	82.1	96.8	98.0
Stern et al. [28]	-	-	-	-	95.7
Saint-Martin et al. [61]	37.5	-	69.0	98.0	-

^a^Results of current work using *coronal* knee MRIs

^b^Results of current work using *sagittal* knee MRIs

^c^Values for the male subjects

### Study limitations and practical implementation

The underlying population of this work was particularly *homogeneous*. Only Caucasian male individuals between 13 and 21 years of age with a middle to high socio-economic status were included. This makes comparison to other studies, e.g., Stern et al., more practicable since multiple factors influencing growth are largely mitigated. One limitation of the developed method is its suitability solely for this specific group. The application on other populations should be validated first. Notwithstanding, it is recommended to perform age estimation on homogeneous groups to attain more reliable results for insufficiently large datasets. Especially, to train separate models for males and females since the latter mature faster and have a different growth pattern [63–66].

Besides gender, the application of the method on individuals with lower socio-economic status has not been evaluated. EASO [10] and Schmeling et al. [67] state that in such a case the method would likely underestimate the subjects since lower socio-economic status generally delays skeletal maturation. This outcome would support the principle of “in dubio pro reo.” This hypothesis could be validated in the future by applying the method on other populations.

The age range in this work did not allow classification on the basis of other age limits such as 14 and 21 years which are relevant legal ages in specific fields. Nonetheless, the focus of this work was the 18-year age limit which is a crucial threshold to separate minors from adults in many countries. The selected age range made it possible to assess this age limit with sufficient margin of error.

A *uniform* age distribution is the optimal starting point for an unbiased age estimation. The coronal dataset was unbalanced but was countered by augmenting the knee MRIs of the subjects from the less populated age groups. A slight bias could still be present when comparing the regression results from DS_cor_ with DS_sag_ which was more uniformly distributed. However, the classification results were superior using DS_sag_ which suggests that a more “favorable” distribution is not necessarily followed by better performance.

In addition to obtaining a uniform age distribution, the size of the dataset in medical applications is often limited due to laws and guidelines to protect the privacy of patients [68]. Although DS_sag_ had over 400 three-dimensional MRIs of the knee, the roughly 40 cases per age group might not be sufficient to fully capture the variability of the group. Furthermore, data-driven methods such as deep learning require a large amount of data to learn a complex task given the high number of unknowns in the system. Cho et al. [68] concluded that 1000 samples per class in the training data is necessary to correctly classify body parts in computed-tomography (CT) images using deep learning. Although it is a different field of study, it gives a perspective of how much data is necessary for data-driven models.

A final limitation of the current study is MRI as image modality due to the high cost in comparison to X-ray due to spacious facilities, equipment, medical technical assistants, and examination duration. In [69–78], a solution is proposed to accelerate acquisition times by reconstructing MRIs using undersampled data in *k*-space. Besides reducing the costs it can lower the stress to patients as well, which is particularly useful for young refugees with traumatic backgrounds. First promising applications of the acceleration technique in the area of age estimation have been analyzed in [71, 74, 75]. Other non-invasive techniques such as ultrasound (US) have yet to be evaluated for their suitability for age estimation, especially when used in combination with machine learning. A recent study by Herrmann et al. [79] proposed a growth plate classification system of the knee for age estimation using US volumes and compared the results with MRI.

## Conclusion

The current work presented a new method for automated age estimation of young individuals based on machine learning—notably CNNs—using MRIs of the knee in coronal and sagittal orientation. The three-step approach first pre-processed the MRIs (correction and standardization), subsequently extracted age-relevant structures (data reduction and simplification), and finally, estimated the chronological age based on the extracted data.

The computer-based method demonstrated its ability to both age regression and majority classification (≥ 18 years) in a fully automated, non-invasive, and user-independent setting.

For the regression task, a combination of a CNN, i.e., AgeNet2D, and a tree-based machine learning algorithm and achieved an MAE of 0.67 ± 0.49 years. Although this was the best-performing model and was evaluated similarly to other studies, it may not capture the full variability of the approach. Therefore, the specifically performed *repeated* stratified *k*-fold validation with a MAE of 0.71 ± 0.55 years may provide a more realistic and reliable estimate of the model’s performance in practice.

For the classification task, AgeNet2D followed by a random forest classifier showed the highest values for sensitivity (88.6%) and specificity (94.2%) and comparable to similar studies. The *repeated* cross-validation results with a sensitivity of 88.4% and a specificity of 86.1% might offer a more realistic and reliable evaluation of the classifier’ performance.

The results of age estimation differed slightly for both imaging planes. While the regression was significantly better using coronal rather than sagittal MRI, the age distribution and size of the datasets were different. For a practical implementation of a similar approach, the imaging plane might have less influence than the factors mentioned above, especially when considering data-driven models that use deep learning, for example. Further analysis could be conducted in the future to evaluate this assumption.
